# Do temperate tree species diversity and identity influence soil microbial community function and composition?

**DOI:** 10.1002/ece3.3313

**Published:** 2017-08-30

**Authors:** Rim Khlifa, Alain Paquette, Christian Messier, Peter B. Reich, Alison D. Munson

**Affiliations:** ^1^ Centre d’étude de la forêt Département des sciences du bois et de la forêt Faculté de foresterie, de géographie et de géomatique Université Laval Québec QC Canada; ^2^ Centre d’étude de la forêt Université du Québec à Montréal Montréal QC Canada; ^3^ Institut des sciences de la forêt feuillue tempérée (ISFORT) Université du Québec en Outaouais Ripon QC Canada; ^4^ Department of Forest Resources University of Minnesota St. Paul MN USA; ^5^ Hawkesbury Institute for the Environment University of Western Sydney Penrith NSW Australia

**Keywords:** belowground ecosystem functioning, biodiversity, IDENT, MicroResp^™^, phospholipid fatty acids, soil microbial community, tree species diversity, tree species identity, TreeDivNet

## Abstract

Studies of biodiversity–ecosystem function in treed ecosystems have generally focused on aboveground functions. This study investigates intertrophic links between tree diversity and soil microbial community function and composition. We examined how microbial communities in surface mineral soil responded to experimental gradients of tree species richness (SR), functional diversity (FD), community‐weighted mean trait value (CWM), and tree identity. The site was a 4‐year‐old common garden experiment near Montreal, Canada, consisting of deciduous and evergreen tree species mixtures. Microbial community composition, community‐level physiological profiles, and respiration were evaluated using phospholipid fatty acid (PLFA) analysis and the MicroResp^™^ system, respectively. The relationship between tree species richness and glucose‐induced respiration (GIR), basal respiration (BR), metabolic quotient (qCO_2_) followed a positive but saturating shape. Microbial communities associated with species mixtures were more active (basal respiration [BR]), with higher biomass (glucose‐induced respiration [GIR]), and used a greater number of carbon sources than monocultures. Communities associated with deciduous tree species used a greater number of carbon sources than those associated with evergreen species, suggesting a greater soil carbon storage capacity. There were no differences in microbial composition (PLFA) between monocultures and SR mixtures. The FD and the CWM of several functional traits affected both BR and GIR. In general, the CWM of traits had stronger effects than did FD, suggesting that certain traits of dominant species have more effect on ecosystem processes than does FD. Both the functions of GIR and BR were positively related to aboveground tree community productivity. Both tree diversity (SR) and identity (species and functional identity—leaf habit) affected soil microbial community respiration, biomass, and composition. For the first time, we identified functional traits related to life‐history strategy, as well as root traits that influence another trophic level, soil microbial community function, via effects on BR and GIR.

## 1 INTRODUCTION

Faced with an accelerated decline in biodiversity and the challenge of maintaining the provision of ecosystem goods and services, the last two decades witnessed a growing number of studies exploring the links between diversity (of different organisms) and ecosystem functions (BEF), with an emphasis on species richness (SR) and on productivity as the measured function. A positive relationship between diversity and productivity was demonstrated in grasslands, aquatic systems, bacterial microcosms, and soil communities (Cardinale et al., 2011, 2012; Hooper et al., 2012; Reich et al., 2012), generally showing a saturating shape indicating functional redundancy (Liang et al., 2016; O'Connor et al., 2017). Several metrics of diversity other than SR may contribute to explain functioning (Hooper et al., 2005), including phylogenetic diversity (Cadotte, Cavender Bares, Tilman, & Oakley, 2009; Paquette, Joly, & Messier, 2015), functional diversity (FD)—defined as the value, range, and distribution of functional traits of organisms in a community (Tilman, Knops, et al., 1997), or community‐weighted mean trait value (CWM; functional mean). Species identity rather than diversity may also control ecosystem functioning (Tobner et al., 2016). However, the different mechanisms by which plant diversity or identity influence ecosystem functioning are still poorly understood, especially in the belowground compartment. As soil microbial communities are major drivers of local and global biogeochemical cycles (Van Der Heijden, Bardgett, & Van Straalen, 2008; Wardle et al., 2004), it is important to investigate by which mechanisms plant species diversity and identity may influence both microbial community structure and functions such as basal respiration.

Soil microbial biomass (glucose‐induced respiration, GIR), microbial activity (basal respiration, BR), and the respiration rate per unit soil microbial biomass—the metabolic quotient (qCO_2_—the efficiency at which the microbial community is functioning; Anderson & Domsch, 1978), are frequently used as indicators of soil quality (Anderson & Domsch, 1993; Zak, Holmes, White, Peacock, & Tilman, 2003), representing measures of function that can be tested along diversity gradients. Soil microbial communities depend on plants for carbon input via litter (leaves, stems, roots) and from rhizodeposition (Hättenschwiler, Tiunov, & Scheu, 2005; Herman, Firestone, Nuccio, & Hodge, 2012). As plant species vary substantially in litter quantity, quality, and root exudate inputs (Mitchell et al., 2012; Vesterdal, Elberling, Christiansen, Callesen, & Schmidt, 2012), these may represent means by which diversity feeds back to the microbial community (Eisenhauer et al., 2017). Species diversity or identity may also indirectly impact microbial communities through microclimatic effects, or via litter inputs or root activity that modify abiotic variables such as soil pH (Eviner & Chapin, 2003).

Previous studies in grasslands generally showed increases in soil microbial biomass and basal respiration in response to increased plant diversity. In the Jena grassland experiment, Eisenhauer et al. (2010) concluded that microbial community functioning (biomass, respiration, and metabolic quotient) was driven by plants species characters, that is, the quality of litter and rhizodeposits (although plant traits were not measured). Bardgett and Shine (1999) observed a decrease in qCO_2_ with the highest diversity of plant litter input in a temperate grassland experiment, supporting the hypothesis that increasing litter diversity increases efficiency of soil biological processes such as decomposition and nutrient turnover (Bardgett & Cook, 1998).

Fewer studies have been undertaken in treed ecosystems. In natural forest, Thoms, Gattinger, Jacob, Thomas, and Gleixner (2010) observed an increase in soil microbial biomass with increasing tree species diversity and suggested that the microbial community is indirectly influenced by aboveground diversity, mostly driven by abiotic factors such soil pH. In a temperate mixed broad‐leaved forest, Scheibe et al. (2015) separated for the first time the effects of tree species diversity and tree identity on soil microbial communities. They observed that tree species identity, especially through leaf traits of the species (notably the C/N ratio of leaf litter) and soil conditions (soil pH), was more important in determining microbial community structure than species richness. However, their study measured only SR and did not include evergreen species, nor mixtures of evergreen and deciduous trees.

We also investigated the effect of species diversity and identity on microbial community structure or composition, using CLPP (community‐level physiological profiles) and PLFA (phospholipid fatty acid analysis). The former provides information about microbial functioning (use of particular C sources) and the latter, microbial community composition. The fungal and bacterial biomass ratio (F:B) is frequently used to assess the state of the decomposer community, showing response of this ratio to plant community composition (Bauhus, Pare, & Cote, 1998; Habekost et al., 2008; Hackl, Pfeffer, Donat, Bachmann, & Zechmeister‐Boltenstern, 2005; Myers, Zak, White, & Peacock, 2001). Fungi are able to decompose recalcitrant organic matter, while bacteria respond rapidly when decomposable C compounds such as sugars, organic, and amino acids are available. For example, Fu et al. (2015) observed that both the aboveground biomass of the understory and the diversity of the tree layer positively influenced the fungal: bacterial ratio in subtropical forests and suggested this effect was related to certain traits or strategies of the aboveground species rather than to changes in diversity per se (Smith et al., 2003). In their study, Thoms et al. (2010) hypothesized that an increase in tree diversity will be correlated with an increasing diversity and abundance of PLFAs in the uppermost soil layer—because the microbial community is affected by litter characteristics such as provision of microhabitats and by nutrient chemistry, the latter having an indirect effect via soil pH. They indeed observed that soils associated with greater leaf litter diversity had the largest total amounts of fatty acids (PLFA), but mainly a common marker for arbuscular mycorrhizal (AM) fungi contributed to this increase. The authors attributed this result to the combination of higher pH values and a higher proportion of *Fraxinus* and *Acer* with AM fungi, which resulted in a direct effect of species on the PLFA.

Although demonstrations of a positive effect of diversity on tree productivity (and other functions) are starting to converge from both experimental and observational studies worldwide (Liang et al., 2016; Zhang, Chen, & Reich, 2012), the mechanisms involved are still poorly understood, especially whether and how they involve other trophic levels than the primary producers (Laforest‐Lapointe, Paquette, Messier, & Kembel, 2017; Tobner, Paquette, Reich, Gravel, & Messier, 2014). In this study, we aimed to go further than previous studies by (1) testing a FD gradient (FD of individual tree functional traits: FDt) in addition to the frequently used SR gradient, to link tree diversity to functioning of another trophic level, that of the surface soil microbial community; (2) testing tree identity effect on the microbial community function and structure, by including a high number of both deciduous and evergreen tree species. We used a 4‐year‐old common garden experiment (IDENT; Tobner et al., 2014) of high‐density tree communities near Montreal, Canada, part of TreeDivNet (Verheyen et al., 2016), consisting of deciduous and evergreen tree species mixtures along orthogonal gradients of SR and FD. The SR gradient was characterized by 12 monocultures, 14 two‐species mixtures, 10 four‐species mixtures, and one mixture containing all 12 species. The a priori FD gradient was achieved by planting several combinations of species at SR levels of 2 and 4 varying in FD, enabling independent tests of FD while holding SR constant. We did not use this gradient in this study. Instead, we tested whether the average values of individual tree traits (CWM) and their variance (FDt; Ricotta & Moretti, 2011) explained potential mechanisms underlying diversity effects.

We hypothesized that:

1. Soil microbial basal respiration and microbial biomass increase with tree SR (following a positive but saturating shape), while respiration rate per unit microbial biomass (qCO_2_) decreases (increased metabolic efficiency in C use);
2. Higher tree diversity SR is associated with an increase in the total amount of PLFAs and in the fungi: bacteria ratio;
3. Soil microbial community functioning and composition are also determined by the identity of trees and functional traits, in particular leaf and root litter chemical traits that are likely to induce changes in soil properties such as C and N (and affecting N availability) and leaf and root bases (K, Mg, Ca) affecting soil pH. Microbial community functioning may also be related to tree community productivity, especially belowground productivity.

## 2 MATERIALS AND METHODS

### 2.1 Site description and experimental design

The site is located at Ste‐Anne‐de‐Bellevue, near Montreal, Québec, Canada, 45.5°N, 73.9°W. Mean annual temperature is 6.2°C with a mean annual precipitation of 963 mm (climate.weatheroffice.gc.ca). The experiment is part of the IDENT network (International Diversity Experiment Network with Trees) that includes seven sites in North America and Europe (Tobner et al., 2014). This study site was established on an agricultural field in spring, 2009, with seedlings of one (deciduous) or two (evergreen) years of age, including 12 early‐ and late – successional North American temperate tree species: *Acer saccharum,* Marsh (Sugar Maple), *Acer rubrum,* L. (Red Maple), *Betula alleghaniensis,* Britton (Yellow Birch), *Betula papyrifera,* Marsh (Paper Birch) and *Quercus rubra,* L. (Northern Red Oak), *Abies balsamea,* (L.) Mill. (Balsam Fir), *Larix laricina,* (Du Roi) K. Koch. (Tamarack), *Pinus strobus,* L. (Eastern White Pine), *Pinus resinosa,* Aiton (Red Pine), *Picea glauca,* Voss (White Spruce), *Picea rubens,* Sarg. (Red Spruce), and *Thuja occidentalis,* L. (Eastern White Cedar). Trees were planted at 50 cm planting distance in square plots of ~4 × 4 m containing 64 individual trees. The design was replicated in 4 blocks, each containing 12 monocultures (SR1), 14 combinations of two‐species mixtures (SR2), 10 combinations of four‐species mixtures (SR4), and one mixture with all 12 species (SR12) for a total of 148 plots used in this study. For the purpose of establishing the experimental design, species mixtures were established to create functional diversity gradients over each of the fixed and independent species richness levels. However, in this study, we did not use this initial a priori FD gradient (please see Tobner et al. 2014, 2016 for in depth discussion of the experimental design). Within plots, trees in mixtures were planted at random with restrictions to prevent the clumping of species. This site is the first tree diversity experiment to have continuously eliminated competing vegetation to isolate the effects of tree SR and FD.

### 2.2 Soil sampling

Soils were sampled in June 2012 (during the fourth growing season), using aluminum cylindrical corers (8.5 cm diameter and 3 cm depth). Two soil cores were randomly sampled per plot from the center of a square created by four trees and then were pooled. Fresh soil samples were sieved at <2 mm, and visible debris was removed before the soils were frozen, prior to MicroResp^™^ and PLFA analysis. Soil texture was assessed by decantation (Bouyoucos, 1962) and soil pH was measured in a prepared 0.01 mol/L CaCl_2_ solution that is added to soil in a 1:2 soil to liquid mixture (Hendershot, Lalande, & Duquette, 1993).

### 2.3 MicroResp^™^ physiological profiles and phospholipid fatty acid extraction (PLFA)

We measured the basal respiration (BR), the glucose‐induced respiration (GIR)—a proxy of the active microbial biomass—the community‐level physiological profiles (CLPP), and PLFA of soil microbial. The individuals that comprise a soil microbial community have different abilities to utilize carbon substrates so that by adding different substrates and measuring respiration, it is possible to obtain a catabolic fingerprint of the community, that is the CLPP (Degens & Harris, 1997). The microbial biomass was determined by GIR (Berard, Bouchet, Sévenier, Pablo, & Gros, 2011; Berard, Mazzia, Sappin Didier, Capowiez, & Capowiez, 2014) based on Anderson and Domsch (1978, 1985) and Chapman, Campbell, & Artz (2007). The CLPP, the BR, and the GIR were measured using the MicroResp^™^ system (Campbell, Chapman, Cameron, Davidson, & Potts, 2003). The procedure of Hamel et al. (2006) was performed for lipid extraction and PLFA analyses (See Appendix S1 for more details). We sampled soils in summer, which is considered the more favorable season to observe compositional shifts using these methods (Grayston, Griffith, Mawdsley, Campbell, & Bardgett, 2001; Thoms & Gleixner, 2013).

### 2.4 Functional diversity and community‐weighted traits calculation

We used an updated list of 17 functional traits for our 12 species, which were measured *in situ* on trees in monocultures between 2011 and 2013 (Table 1, and Appendix S2 and Table S2), with the exception of wood density and seed mass, which were obtained from the literature. We chose the traits based on demonstrated importance for positive mixture effects on tree productivity: wood density (Swenson & Enquist, 2007); seed mass (Ben‐Hur, Fragman‐Sapir, Hadas, Singer, & Kadmon, 2012), maximum height (Paquette & Messier, 2011), specific root length, fine root diameter, and branching intensity (Tobner et al., 2014), or on litter decomposition: leaf litter C and N concentrations (Jewell, 2013). We included measured root chemistry traits (macronutrients) which are also likely to feed back to soil microbial communities (Khlifa et al. in preparation). Functional dispersion indices were then calculated (Laliberté & Legendre, 2010; Laliberté, Legendre, & Shipley, 2014) for each trait (FDt), to identify the relative importance of each on BR and GIR (Table 1). The average values of functional traits (CWM)—functional mean—were calculated for each community (plot) as the product of the average trait value of a species and its relative abundance in the community, averaged across all species (Lavorel et al., 2008). Both CWM traits and functional variation (FDt) were weighted by species‘ relative abundance in the community at the end of season 2012.

**Table 1:** Effect of individual trait means (CWM) and variances (FDt) on soil microbial basal respiration (BR) and soil microbial biomass (GIR)

Functional Trait	BR		GIR	
	FDt	CWM	FDt	CWM
Litter nitrogen concentration	0.025↗	0.436	0.722	0.004↗
Litter carbon concentration	0.952	0.025↘	0.316	0.027↘
Leaf dry matter content	0.936	0.440	0.222	0.067
Specific leaf area	0.173	<0.001↗	0.630	<0.001↗
Root diameter	0.647	0.018↗	0.038↘	<0.001↘
Root branching intensity	0.687	0.021↗	0.490	<0.001↗
Specific root length	0.895	0.003↗	0.043↘	<0.001↗
Root nitrogen concentration	0.704	0.393	0.429	0.009↘
Root carbon concentration	0.901	0.329	0.082	<0.001↗
Root phosphorus concentration	0.784	0.259	0.420	0.042↘
Root potassium concentration	0.935	0.184	0.314	0.184
Root calcium concentration	0.019↗	0.496	0.350	0.256
Root magnesium concentration	0.998	0.662	0.979	0.253
Tree height	0.603	0.004↗	0.507	<0.001↗
Ground diameter	0.322	0.023↗	0.483	<0.001↗
Wood density	0.309	0.005↗	0.069	<0.001↗
Seed mass	0.576	0.831	0.007↗	0.047↗

The values presented are the *p* values of mixed model regressions, and significant positive or negative effects (at α = 0.05) are represented with arrows.

### 2.5 Statistical analyses

All statistical analyses were carried out in the R environment (version 3.2.1 R Core Team 2015). Linear mixed‐effect models were developed using the *lme* function in the *nlme* package (Pinheiro, Bates, DebRoy, & Sarkar, 2007), to test the effect of SR (*n* = 148), FD (*n* = 96; monocultures removed), FDt or CWM, as a fixed factors, on microbial parameters (BR, GIR, qCO_2_), PLFA groups, and soil properties as response variables with plot identity (to take into account an unbalanced design). The blocks were included as random factors and soil % clay as an environmental covariable. Mean separations between SR levels were applied using Tukey's HSD tests with the *glht* function in the *multcomp* package (Hothorn, Bretz, & Westfall, 2008). Predicted values and standard errors of the mixed models were computed using the *perdictSE* function in the *AICcmodavg* package (Mazerolle, 2016). In all linear mixed‐effect models, the normality of residuals and homogeneity of variance were tested and transformations were carried out when necessary. Statistical significance was set at the 0.05 level.

Correlations between tree functional traits and microbial parameters (GIR and BR), and above‐/belowground productivity and the same microbial parameters were tested using the *cor.test* function. Aboveground productivity of plots was measured in 2013 (volume increment from Tobner et al., 2016), while annual fine root productivity was measured by ingrowth cores in 2012–2013 (Khlifa et al. in preparation).

The shape of the relationship between soil microbial parameters (BR, GIR, qCO_2_) and tree SR was examined through the fitting of five different functions (linear, exponential, logarithmic, power, and Michaelis–Menten (M–M) (Cardinale et al., 2011; Delgado‐Baquerizo et al., 2016; Reich et al., 2012). We selected the best model fits by following Akaike information criteria (AICc; Burnham and Anderson 2003). Here, we consider a ΔAICc >2 threshold (Burnham & Anderson, 2003) to differentiate between substantially different models.

The CLPP datasets were standardized by scaling (subtracting the mean SIR of all soils on all substrates, then dividing by the standard deviation) and then subjected to principal component analysis (PCA) followed by a between‐class analysis to discriminate among SR levels (Chessel, Thioulouse, & Jean, 2004). We also used a PCA to characterize tree species monocultures by their C source utilization (CLPP). The PCA was computed using the *dudi.pca* function of the *ade4* package (Dray & Dufour, 2007).

## 3 RESULTS

### 3.1 Tree species richness and microbial community parameters and composition

**Figure 1:**
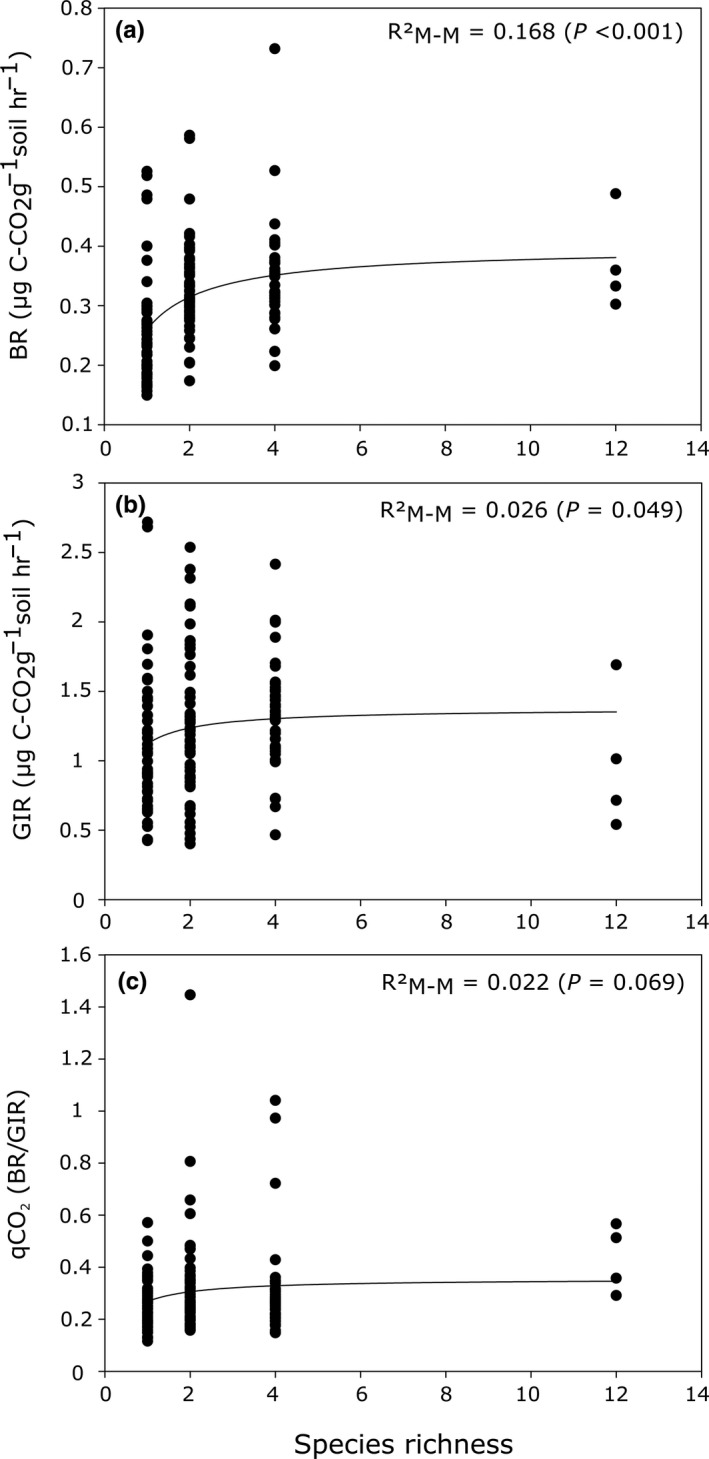
Relationship between soil microbial parameters and tree species richness. (A) BR (basal respiration), (B) GIR (active microbial biomass) expressed in μg C‐CO_2_ g^−1^ soil hr^−1^, (C) qCO_2_ (metabolic quotient; *n* = 4 replications for each identity plot for a total of 148 plots). The solid lines represent fitted regressions for the best model

**Figure 2:**
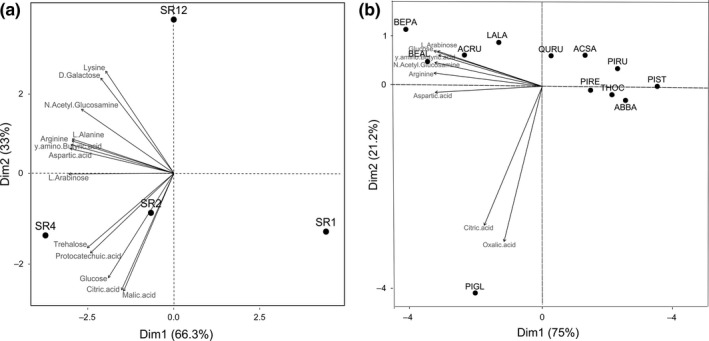
(a) Biplot of the principle components analysis based on the community‐level physiological profiles (CLPP) measurements of the different soils sampled: ordination of four tree species richness levels, and correlation plot between the PCA axes and the 13 different substrates catabolized that best explained the variations. (b) Biplot of the principle components analysis based on CLPP measurements of the different soils sampled: ordination of twelve tree monocultures, and correlation plot between the PCA axes and the eight different substrates catabolized that best explained the variations. SR1 (monocultures), SR2 (two‐species mixtures), SR4 (four‐species mixtures), and SR12 (all twelve species together). ABBA (*Abies balsamea*)*,* ACRU (*Acer rubrum*)*,* ACSA (*Acer saccharum*)*,* BEAL (*Betula alleghaniensis*)*,* BEPA (*Betula papyrifera*)*,* LALA (*Larix laricina*)*,* PIGL (*Picea glauca*)*,* PIRU (*Picea rubens*)*,* PIRE (*Pinus resinosa*)*,* PIST (*Pinus strobus*)*,* QURU (*Quercus rubra*)*,* THOC (*Thuja occidentalis*)

The Michaelis–Menten (M–M) function appeared as the best model shaping the relationship between tree SR and BR. However, the models could not distinguish among M–M, logarithmic and power functions for GIR, neither among the five tested functions for qCO_2_ (see Appendix S2; Table S4). As M–M was the best model for BR and was either the best or close to the best (but not distinguishable) for GIR and qCO_2_, we used the M–M function for describing the relationship between all three soil microbial parameters and tree SR (Figure 1). The PCAs conducted on the 15 different C sources measured on all SR levels revealed a clear separation of monocultures from mixtures (Monte Carlo tests; *p *=* *.001) (first two axes accounting for 99.3% of the total variability, Figure 2a). Dim1 (66.3% of total variability) appeared to separate SR1 from the other SR levels, demonstrating that monocultures are characterized by lower catabolic activity, whereas Dim2 (33% of total variability) appeared to separate SR12 from SR2 and SR4 mixtures. The number of the C sources catabolized was higher in SR4 mixtures.

SR did not influence total PLFA (PLFA_tot_; *p *=* *.836), PLFA for different taxonomic groups (fungi (PLFA_fun_; *p *=* *.781), bacteria (PLFA_bact_; *p *=* *.632), actinobacteria (PLFA_act_; *p *=* *.06), Gram‐positive (PLFA_pos_; *p *=* *.633), and Gram‐negative bacteria (PLFA_neg_; *p *=* *.935); neither was the fungi: bacteria ratio affected by SR (*p *=* *.389) (Appendix S2; Table S3).

### 3.2 Functional diversity and microbial community parameters and composition

We tested the variance of each functional trait (FDt) separately (in a full model adjusted for SR levels) and found a positive effect of root calcium and litter nitrogen on BR (Table 1). We also found a positive effect of variance in seed mass and a negative effect of variance in root diameter and specific root length on GIR. The variance in wood density and root carbon also had a negative effect on GIR although these results did not obtain significance at the 5% threshold (Table 1).

The CWM influenced BR and GIR (Table 1); GIR was affected by a greater number of traits than BR. Both parameters were positively related to the CWM of specific leaf area, root branching intensity, root‐specific length, tree height, tree ground diameter and wood density, and negatively related to litter carbon content. BR was positively related with root diameter, while GIR was negatively correlated with the same trait.

### 3.3 Identity effects and microbial community parameters and composition

The PCAs on CLPP measurements for the 12 monocultures discriminated evergreen species from deciduous tree species (first two axes accounted for >96% of total variability, Figure 2b). The first axis (75% of total variability) showed that a higher number of catabolized C sources were used by microbial communities associated with deciduous mixtures, compared to evergreen species. Dim2 (21.2% of total variability) revealed that microbial communities associated with *Picea glauca* (Figure 2b) appeared to use dominantly carboxylic acid substrates (oxalic and citric acids), compared to C sources used by other monocultures. Moreover, evergreen species were characterized by lower values of GIR (*p *<* *.001) and BR (*p *=* *.010) compared to deciduous (data not shown). Globally, species identity (monocultures) did not influence microbial community structure (no differences in individual PLFAs) with the exception of *Larix laricina* which showed significantly higher amounts of total PLFAs and PLFAs for total bacteria than *Thuja occidentalis* and higher amounts of PLFAs for Gram‐positive bacteria than *Thuja occidentalis, Picea glauca* and *Picea rubens* (Appendix S2; Table S3).

There were no correlations between the ratio of plantation aboveground/belowground productivity and microbial parameters (GIR and BR; *r* = .197, *p = *.178 and *r* = .047, *p = *.750, respectively), neither between belowground productivity and GIR (*r* = −.195, *p = *.183) and BR (*r* = −.164, *p = *.263). However, aboveground productivity was significantly correlated with GIR (*r* = .386, *p = *.006) and with BR (*r* = .268, *p *=* *.065) (data not shown).

## 4 DISCUSSION

Overall, we observed that the function (biomass, respiration, efficiency, use of carbon sources) of the soil microbial community responded to species richness (SR), but that the structure and composition did not (Phospholipid fatty acid extraction, PLFA). Both microbial biomass (GIR) and respiration (BR) responded to the community‐weighted mean (CWM) of individual tree traits, or to a lesser degree to trait variance (FDt), mainly to traits related to life‐history strategy (fast or slow growing) or to litter chemical traits. In addition, GIR (especially) and BR were both positively related to aboveground tree productivity.

The relationship between soil microbial biomass, basal respiration, and efficiency and tree SR followed a positive, saturating shape, indicating redundancy (Cardinale et al., 2011). Functional redundancy suggests that some fraction of tree species can be lost with minimal effects on microbial community functioning. However, redundancy may not be assured if the function is related to certain single species that use resources in a unique way (Cardinale, Ives, & Inchausti, 2004; Tilman, Lehman, & Thompson, 1997). In the Jena grasslands (Lange et al., 2015), higher microbial activity was linked to a greater soil C storage, especially of more stable C; however, we did not observe either increased soil total C or N (or size fractions) on our study site (Khlifa et al. in preparation). Contrary to expectations, the qCO_2_—which is often used as an ecophysiological indicator of stress (Anderson, 2003)—increased rather than decreased with increasing SR levels (*R* ^2^ weak), indicating that the associated microbial communities were not more efficient in carbon use with increasing tree diversity (Anderson & Domsch, 1993). This contrasts with previous findings (Bardgett & Shine, 1999; Eisenhauer et al., 2010). While efficiency did not increase, the number of carbon sources being used by microbes was generally greatest in SR4 (Figure 2a), which may indicate greater complementarity of carbon use by the more diverse community, contributing to increased microbial biomass and respiration observed. The lack of response of microbial community structure to tree SR was also observed in boreal (Lucas‐Borja, Candel Pérez, López Serrano, Andrés, & Bastida, 2012) and temperate broad‐leaved forests (Scheibe et al., 2015), while modifications to structure were observed in manipulated diversity gradients in grasslands (Eisenhauer et al., 2010; Scherber et al., 2010) as well as in other tree diversity studies (Fu et al., 2015; Thoms et al., 2010). Numerous studies highlighted the importance of abiotic factors such as soil pH, soil texture and soil moisture (Brockett, Prescott, & Grayston, 2012; Merilä et al., 2010; Wu et al., 2012) or tree species identity (Scheibe et al., 2015) as drivers of structure, rather than changes in tree species richness. In our study, abiotic variables (soil pH, soil texture) did not differ among SR levels nor among tree species (Appendix S2; Table S1). These abiotic feedbacks may take longer than the 4 years of the current study; many of the studies observing abiotic effects were either natural forests or longer‐term experiments (Brockett et al., 2012; Merilä et al., 2010; Wu et al., 2012).

In response to the third hypothesis, we did observe identity effects on microbial community function, principally a difference between deciduous and evergreen species. Microbial communities associated with deciduous were characterized by higher values of biomass (GIR) and respiration (BR) compared with those of evergreen tree species. This difference between these two plant groups was also observed for both above‐ and belowground productivity (former higher for deciduous, latter higher for evergreen species; Khlifa et al. in preparation; Tobner et al., 2016; Archambault, 2016). On the same study site, Rivest, Paquette, Shipley, Reich, and Messier (2015) observed higher BR of the soil microbial community in *Larix laricina* monocultures (highest growth rate) compared to *Acer saccharum* monocultures. We suggest, as the latter authors did, that higher aboveground productivity promotes higher carbon inputs (and diversity of inputs, as observed), contributing to both higher microbial biomass (GIR) and activity (BR); the measured positive correlations between these parameters (especially GIR) and aboveground productivity support this hypothesis. Few studies have reported this latter relationship, but in wheat production systems, Tautges, Sullivan, Reardon, and Burke (2016) noted that wheat yield was correlated with soil fungal and bacterial abundance, measured by QPCR (quantitative polymerase chain reaction analyses) after DNA extraction. We also observed that deciduous tree species were generally able to utilize a wider spectrum of organic C compounds than evergreen tree species (Figure 2b), promoting complementarity of C use in mixtures. Microbial communities associated with *Betula papyrifera* and *Betula alleghaniensis* appeared to be able to metabolize the highest number of C sources, likely due to life strategy of these species which is characterized by an easier decomposability of their leaf and root organic inputs. This species effect might eventually also contribute to more stable C storage, as noted by Lange et al. (2015) with greater microbial activity and C processing. Contrary to the lack of effect of SR on microbial composition, we did observe minor shifts in PLFA composition with tree species; only *Larix laricina* showed a higher abundance of total PLFAs, and PLFAs for total bacteria, due to a higher amount of Gram‐positive bacteria compared to other species (see Appendix S2; Table S3). Again, the fact that this is the highest aboveground producer (Tobner, 2014) may contribute to this first observed effect of identity on community structure. Microbial community structure then may respond more rapidly to changes in quantity rather than changes in quality of inputs. Furthermore, Rivest et al. (2015) observed lowest soil moisture under *Larix* monocultures, indicating (as above) that microclimate or other abiotic factors may be most important as feedback to microbial community structure.

Our study took an important step in using individual traits (community mean–CWM and variance –FDt) to aid in the identification of mechanisms responsible for changes in microbial community function. Many of the same functional traits affected both GIR and BR, although there were a few differences. Both functions were sensitive to the life‐history strategies of trees (SLA, tree height, diameter, and wood density) as well as a number of root traits (root branching intensity, specific root length) associated with higher resource capture potential. All of the life‐history traits showed positive relationships, again underlining a relationship of microbial biomass and respiration to a fast‐growing strategy of trees. Both root traits were also positively related to the two parameters, indicating that an exploitative mode of root growth is related to higher microbial biomass and activity, possibly through greater rhizodeposition inputs. In both cases, lower C concentration of leaf litter (easier decomposability) was also linked to higher microbial biomass and activity. These results suggest some potential mechanisms of the feedback of traits on microbial community function: quantity of inputs, rhizodeposition, and litter quality (Eisenhauer et al., 2017). The microbial biomass seemed more responsive to root chemical quality than does respiration (no significant signals). Microbial biomass (GIR) was also generally more strongly related to CWM than was BR, suggesting that microbial biomass depends more on species identity or leaf habit. In general, CWM had a greater effect on microbial community functioning (GIR and BR) compared to FDt; a greater number of traits influenced through CWM and relationships were generally stronger. Recent studies on the same site showed that CWM better explained tree productivity and diversity effects than did FD (Tobner et al., 2016). Similarly, Jewell (2013) observed that for early stages of leaf litter decomposition, CWM of leaf traits was more important than FD, while surface soil respiration increased with increasing tree FD, independently of average trait values. These cumulated results (with our own) on the same site indicate that at this stage of stand development, the importance of certain traits of dominant species has more effect on ecosystem processes—in our case microbial community functions (GIR and BR)—than functional diversity (Garnier, Navas, & Grigulis, 2016). Results also underline the importance of tree species identity for ecosystem functioning and suggest potential negative effects of the loss of key tree species, or particular plant groups such as evergreen or deciduous tree species to microbial functions, and therefore processes such as decomposition and nutrient turnover.

## 5 CONCLUSIONS

This is the first tree diversity experiment to isolate the effects of tree species richness (SR) and functional diversity (FD) on soil microbial community function (GIR—a proxy of microbial biomass—BR and qCO_2_). Increasing tree SR affected all three soil microbial community parameters compared to monocultures. Although microbial community composition (PLFA) did not generally respond to SR, a modest response to tree identity was observed for the most rapidly growing species: *Larix laricina*. Microbial communities associated with deciduous tree species generally metabolized a higher number of C sources compared to evergreen tree species, while S4 mixtures were also associated with a greater diversity of C sources. This could indicate a mechanism of complementarity; including both deciduous and evergreen tree species in mixtures may increase and diversify the resources available to microbes, thereby contributing to higher soil microbial biomass (productivity) and activity (respiration). Significantly, we noted that aboveground productivity was a potential driver of belowground microbial biomass. Some of the mechanisms involved in these responses are identified via individual trait analyses: faster growth of trees (greater inputs), improved exploitation by roots (higher SLR and root branchiness), and improved litter quality (higher leaf litter N and lower C). We note that CWM of tree traits is often more important than its FDt (functional variance) to GIR and BR, supporting more strongly the importance of dominance (and biomass) effects on microbial processes at this early stage of forest succession. Our results suggest that after only 4 years, both tree species richness and identity influence soil microbial functioning and composition in young tree communities. Overall, these results represent an important advance in our comprehension of the intertrophic relationships between tree and soil microbe communities in these planted forest ecosystems.

## CONFLICT OF INTEREST

There are no conflicts of interest to declare.

## AUTHORS’ CONTRIBUTIONS

The experimental design was initiated and established by C.M. and A.P., R.K., and A.D.M. developed the hypotheses, designed methodology, and collected the data; R.K. analyzed the data; R.K. and A.D.M. led the writing of the manuscript. All authors contributed critically to the drafts and gave final approval for publication.
