# Supplementary material for: Do temperate tree species diversity and identity influence soil microbial community function and composition?
Source: Ecol Evol. 2017 Aug 30;7(19):7965–74. doi: 10.1002/ece3.3313 (PMC5632628; doi:10.1002/ece3.3313)
Supplement: Supplementary file 1 [file ECE3-7-7965-s001.docx]

**Supporting Information**

Additional Supporting Information may be found in the online version of this article:

**Appendix S1.** Supplementary methods.

**Appendix S2.** Tables.

**Table S1.** Soil characteristics under tree species mixtures and their identities at the IDENT Montreal site (Québec. Canada).

**Table S2.** Mean values and units of tree functional traits used to compute functional diversity indices (FD_t_) and community weighted means (CWM).

**Table S3.** Soil microbial community composition under tree species mixtures and their identities at the IDENT Montreal site (Québec. Canada).

**Table S4**. Model fit statistics and AICc index for the different functions describing the relationship between soil microbial parameters and tree species richness.

**Appendix S1. Supplementary methods.**

**MicroResp^TM^ physiological profiles.** The MicroResp™ method allows soil respiration and microbial community physiological profiles to be determined colorimetrically in microplates (Campbell *et al.* 2003), in a wide range of applications (Tlili *et al.* 2011; Sassi *et al.* 2012). In the present study, the carbon sources used were L-alanine, L-arabinose, L-arginine, citric acid, D-fructose, D-galactose, D-glucose, L-malic acid, L-lysine, N-acetyl glucosamine, oxalic acid, protocatechuic acid, DL-aspartic acid, γ-aminobutyric acid and D-trehalose. Soil moisture content of all samples was pre-adjusted to 40% of the water holding capacity (WHC), which is in the optimal range of WHC for microbial respiration (Ilstedt, Nordgren & Malmer 2000; Moreno *et al.* 2002; Campbell *et al.* 2003). Soils were then pre-incubated for one week in microcosms at ambient temperature (23°C ± 2), in dark conditions (Berard *et al.* 2014). Community Level Physiological Profiles (CLPP) and basal respiration (BR) were measured using the MicroResp™ system (Campbell *et al.* 2003). The system consists of a detection microplate, containing 150 ml purified agar (1%), cresol red indicator dye (12.5 µg ml^-1^), potassium chloride (150 mM) and sodium bicarbonate (2.5 mM), attached to a 96-deep-well microplate (1.2 ml volume). The microplate is filled with ~ 0.35 g of fresh soil weight per well; only water is added to wells (25 µl) for BR measurements, or one of 15 different C sources are added, based on their ecologically relevance to soil and root ecology (Campbell, Grayston & Hirst 1997).Carbon sources were applied in 25 ml to achieve a final concentration of 30 mg g^-1^ soil water for Substrate-induced respiration (SIR) measurements. The carbon sources used were L-alanine, L-arabinose, L-arginine, citric acid, D-fructose, D-galactose, D-glucose, L-malic acid, L-lysine, N-acetyl glucosamine, oxalic acid, protocatechuic acid, DL-aspartic acid, γ-aminobutyric acid and D-trehalose.

All measures were done in triplicate and with two soil samples per plate after 6 h incubation in the dark at room temperature (23 ± 1°C). The absorbance of the detection microplate was measured at 570 nm (Biotek EL-800 spectrophotometer). The average amount of CO_2_ that evolved per sample was calculated and used to normalize individual C source concentrations before multivariate analysis (Campbell *et al.* 2003). A calibration curve of absorbance versus head space equilibrium CO_2_ concentration (measured by gas chromatography) was fitted to a regression model, and results were expressed in µg C-CO_2_ g^−1^soil h^−1^ (Campbell *et al.* 2003; Berard *et al.* 2011, 2014). Microbial biomass was determined by glucose-induced respiration (GIR) (Berard *et al.* 2011, 2014) based on Anderson & Domsch (1978, 1985) and (Chapman, Campbell & Artz 2007). The respiration rate per unit soil microbial biomass - the metabolic quotient qCO_2_ - is the ratio of basal respiration (BR) to microbial biomass (GIR) (Saul-Tcherkas & Steinberger 2009; Berard *et al.* 2011, 2014), and is a measure of the ecophysiological status of soil microorganisms (Anderson & Domsch 2010). It has been used in a large number of studies and for different purposes, for example, microbe status in relation to plant succession (Insam & Haselwandter 1989) or to metal soil contamination (Berard *et al.* 2014).

**Phospholipid fatty acid extraction (PLFA).** The determination of the phospholipid fatty acid (PLFA) pattern of soil organisms is one of the most commonly used methods to represent living soil microbial biomass and to identify the presence or absence of various functional groups of interest through known PLFA biomarkers (Frostegård, Tunlid & Bååth 2011). The procedure of Hamel *et al.* (2006) was performed for lipid extraction and PLFA analyses. Briefly, total soil lipids were extracted by shaking about 2 g of freeze-dried soil in a dichloromethane-methanol-citrate buffer mixture (1:2:0.8) for 1 h. The phospholipids were separated from other lipids on a silicic acid column and esterified forming fatty acid methyl esters (FAME) which were separated and quantified by gas chromatography (Findlay 2004).Gas chromatography was carried out on an Agilent 7890A equipped with a 7693 Autosampler and a flame ionisation detector; the carrier gas used was hydrogen. PLFA identities were assigned based on comparison of retention times to known standards. Fatty acids were named according to the ω-designation described as follows: total number of carbons followed by a colon; the number of double bonds; the symbol ω; the position of the first double bond from the methyl end of the molecule. The biomarkers used to discriminate different PLFA are commonly used for microbial community profiling (Leckie 2005).The abundance of individual PLFAs was expressed as ng PLFA g^-1^ dry soil.

**Fine root chemistry (root traits).** Fine roots (≤ 2mm diameter) were sampled in October 2012 in a central point between four trees to maximise species interactions among local tree neighbourhoods. Five and three soil cores were sampled per plot in mixed communities and monocultures, respectively, to a depth of 15 cm. The fine roots were then rinsed, dried in a forced-air oven at 65°C to constant weight, then finely ground using a ball mill apparatus (Retsch mixer mill, MM200; Retsch GmbH, Haan, Germany). For oot chemistry, C was determined by dry combustion (Trumac CNS, LECO Corp., MI, USA) and the concentrations of nitrogen (N), phosphorus (P), potassium (K), calcium (Ca) and magnesium (Mg) were determined by digestion (Parkinson and Allen 1975) followed by atomic absorption spectroscopy (Optima 4300 DV ICP-OES, Perkin-Elmer Inc., Waltham, MA, USA).

**References**

Anderson, J.P.E. & Domsch, K.H. (1978) A physiological method for the quantitative measurement of microbial biomass in soils. *Soil Biology and Biochemistry*, **10**, 215–221.

Anderson, T.H. & Domsch, K.H. (1985) Determination of ecophysiological maintenance carbon requirements of soil microorganisms in a dormant state. *Biology and Fertility of Soils*, **1**, 81–89.

Anderson, T.H. & Domsch, K.H. (2010) Soil microbial biomass: The eco-physiological approach. *Soil Biology and Biochemistry*, **42**, 2039–2043.

Berard, A., Bouchet, T., Sévenier, G., Pablo, A.-L.L. & Gros, R. (2011) Resilience of soil microbial communities impacted by severe drought and high temperature in the context of Mediterranean heat waves. *European Journal of Soil Biology*, **47**, 333–342.

Berard, A., Mazzia, C., Sappin Didier, V., Capowiez, L. & Capowiez, Y. (2014) Use of the MicroResp^TM^ method to assess pollution-induced community tolerance in the context of metal soil contamination. *Ecological Indicators*, **40**, 27–33.

Campbell, C.D., Chapman, S.J., Cameron, C.M., Davidson, M.S. & Potts, J.M. (2003) A rapid microtiter plate method to measure carbon dioxide evolved from carbon substrate amendments so as to determine the physiological profiles of soil microbial communities by using whole soil. *Applied and Environmental Microbiology*, **69**, 3593–3599.

Campbell, C.D., Grayston, S.J. & Hirst, D.J. (1997) Use of rhizosphere carbon sources in sole carbon source tests to discriminate soil microbial communities. *Journal of Microbiological Methods*, **30**, 33–41.

Chapman, S.J., Campbell, C.D. & Artz, R.R.E. (2007) Assessing CLPPs using MicroResp^TM^. *Journal of Soils and Sediments*, **7**, 406–410.

Findlay, R.H. (2004) Determination of microbial community structure using phospholipid fatty acid profiles. *Molecular Microbial Ecology Manual, 2nd Edition. Kluwer Academic Publishers, Netherlands*, pp. 983–1004.

Frostegård, Å., Tunlid, A. & Bååth, E. (2011) Use and misuse of PLFA measurements in soils. *Soil Biology and Biochemistry*, **43**, 1621–1625.

Hamel, C., Hanson, K., Selles, F., Cruz, A.F., Lemke, R., McConkey, B. & Zentner, R. (2006) Seasonal and long-term resource-related variations in soil microbial communities in wheat-based rotations of the Canadian prairie. *Soil Biology and Biochemistry*, **38**, 2104–2116.

Ilstedt, U., Nordgren, A. & Malmer, A. (2000) Optimum soil water for soil respiration before and after amendment with glucose in humid tropical acrisols and a boreal mor layer. *Soil Biology and Biochemistry*, **32**, 1591–1599.

Insam, H. & Haselwandter, K. (1989) Metabolic quotient of the soil microflora in relation to plant succession. *Oecologia*, **79**, 174–178.

Leckie, S.E. (2005) Methods of microbial community profiling and their application to forest soils. *Forest Ecology and Management*, **220**, 88–106.

Moreno, J.L., Hernández, T., Pérez, A. & Garcı́a, C. (2002) Toxicity of cadmium to soil microbial activity: effect of sewage sludge addition to soil on the ecological dose. *Applied Soil Ecology*, **21**, 149–158.

Sassi, M. Ben, Dollinger, J., Renault, P., Tlili, A. & Bérard, A. (2012) The FungiResp method: An application of the MicroResp^TM^ method to assess fungi in microbial communities as soil biological indicators. *Ecological Indicators*, **23**, 482–490.

Saul-Tcherkas, V. & Steinberger, Y. (2009) Substrate utilization patterns of desert soil microbial communities in response to xeric and mesic conditions. *Soil Biology and Biochemistry*, **41**, 1882–1893.

Tlili, A., Marechal, M., Montuelle, B., Volat, B., Dorigo, U. & Bérard, A. (2011) Use of the MicroResp^TM^ method to assess pollution-induced community tolerance to metals for lotic biofilms. *Environmental Pollution*, **159**, 18–24.

Tobner, C.M. (2014) Traits fonctionnels des arbres - de la plasticité intraspécifique aux effets de leur diversité sur le fonctionnement de l'écosystème. *Ph.D. thesis, Université du Québec à Montréal, Montréal, QC.*

**Appendix S2. Tables**

**Table S1.** Soil characteristics under tree species mixtures and their identities at the IDENT Montreal site (Québec. Canada). The numbers are mean values. SR1 (monocultures), SR2 (two species mixtures), SR4 (four species mixtures) and SR12 (all twelve species together). Ab (*Abies balsamea*)*,*Ar (*Acer rubrum*)*,* As (*Acer saccharum*)*,* Ba (*Betula alleghaniensis*)*,* Bp (*Betula papyrifera*)*,* Ll (*Larix laricina*)*,* Pg (*Picea glauca*)*,* Pru (*Picea rubens*)*,* Pre (*Pinus resinosa*)*,* Ps (*Pinus strobus*)*,* Qr (*Quercus rubra*)*,* To (*Thuja occidentalis*)*.*

| **Treatment** | **SR**  **levels** | **FDclass**  **levels** | **pH** | **Clay %** | **Sand %** |
| --- | --- | --- | --- | --- | --- |
| **SR1** | 1 | 0 | 5.40 | 10.1 | 86.5 |
| **SR2** | 2 | - | 5.40 | 10.4 | 86.3 |
| **SR4** | 4 | - | 5.38 | 11.4 | 85.4 |
| **SR12** | 12 | - | - | - | - |
| **Ab** | 1 | 0 | 5.43 | 23.5 | 71.8 |
| **Ar** | 1 | 0 | 5.29 | 17.4 | 76.9 |
| **As** | 1 | 0 | 5.23 | 8.0 | 90.9 |
| **Ba** | 1 | 0 | 5.33 | 4.2 | 93.0 |
| **Bp** | 1 | 0 | 5.59 | 9.8 | 86.67 |
| **Ll** | 1 | 0 | 5.32 | 10.8 | 86.0 |
| **Pg** | 1 | 0 | 5.79 | 6.9 | 90.8 |
| **Pre** | 1 | 0 | 5.22 | 8.9 | 87.8 |
| **Pru** | 1 | 0 | 5.44 | 11.3 | 85.2 |
| **Ps** | 1 | 0 | 5.33 | 2.5 | 94.8 |
| **Qr** | 1 | 0 | 5.33 | 12.8 | 82.2 |
| **To** | 1 | 0 | 5.49 | 5.5 | 92.3 |
| **PrePru** | 2 | 1 | 5.39 | 4.3 | 93.0 |
| **LlPs** | 2 | 2 | 5.47 | 5.7 | 91.3 |
| **BaQr** | 2 | 3 | 5.39 | 10.5 | 87.3 |
| **BpQr** | 2 | 4 | 5.32 | 5.2 | 90.8 |
| **PgPs** | 2 | 5 | 5.43 | 12.6 | 83.2 |
| **AbAr** | 2 | 6 | 5.23 | 16.4 | 79.4 |
| **AsLl** | 2 | 7 | 5.60 | 9.5 | 87.2 |
| **AsTo** | 2 | 8 | 5.42 | 16.6 | 79.2 |
| **ArBa** | 2 | 2 | 5.46 | 7.3 | 91.2 |
| **PrePs** | 2 | 2 | 5.43 | 7.7 | 89.8 |
| **LlPg** | 2 | 4 | 5.43 | 8.6 | 88.9 |
| **BpPs** | 2 | 5 | 5.32 | 10.3 | 87.0 |
| **AbAs** | 2 | 7 | 5.44 | 20.4 | 75.4 |
| **ArTo** | 2 | 7 | 5.34 | 11.0 | 85.2 |
| **AbPgPrePru** | 4 | 1 | 5.42 | 5.2 | 91.7 |
| **ArBaBpQr** | 4 | 2 | 5.46 | 5.5 | 91.7 |
| **BaPrePruPs** | 4 | 3 | 5.37 | 11.4 | 84.9 |
| **AbBpLlPg** | 4 | 4 | 5.39 | 9.7 | 87.7 |
| **AbAsPruPg** | 4 | 5 | 5.18 | 17.7 | 78.0 |
| **LlPsQrTo** | 4 | 6 | 5.35 | 13.1 | 83.9 |
| **ArQrPsTo** | 4 | 7 | 5.31 | 7.8 | 89.7 |
| **AsBaPgTo** | 4 | 8 | 5.49 | 11.0 | 86.2 |
| **PrePsPruPg** | 4 | 2 | 5.40 | 11.5 | 86.4 |
| **BpPsAsPg** | 4 | 7 | 5.40 | 21.4 | 73.6 |

| **Functional**  **traits** | **Units** | **Species** | | | | | | | | | | | |  |
| --- | --- | --- | --- | --- | --- | --- | --- | --- | --- | --- | --- | --- | --- | --- |
|  |  | **Ab** | **Ar** | **As** | **Ba** | **Bp** | **Ll** | **Pg** | **Pre** | **Pru** | **Ps** | **Qr** | **To** | **Sources** |
| **Litter nitrogen** | mg N/N | 14.84 | 6.87 | 6.91 | 12.16 | 13.18 | 6.91 | 7.53 | 5.74 | 10.48 | 7.43 | 9.11 | 8.59 | (1) |
| **Litter carbon** | mg C/C | 514.55 | 460.78 | 435.42 | 479.88 | 481.29 | 511.62 | 491.50 | 519.70 | 510.52 | 525.40 | 466.64 | 498.79 | (1) |
| **Leaf dry matter content** | g/g | 414.35 | 427.34 | 411.87 | 325.57 | 287.20 | 295.36 | 408.93 | 344.20 | 408.06 | 343.84 | 424.20 | 274.50 | (1) |
| **Secific leaf area** | m^2^/g | 6.23 | 11.85 | 11.27 | 13.34 | 13.09 | 8.26 | 4.35 | 4.54 | 4.68 | 6.54 | 9.58 | 5.36 | (1) |
| **Root diameter** | mm | 0.45 | 0.35 | 0.33 | 0.28 | 0.26 | 0.38 | 0.33 | 0.37 | 0.27 | 0.56 | 0.27 | 0.57 | (2) |
| **Root branching intensity** | N°of root tips/cm | 1.90 | 3.10 | 2.70 | 4.00 | 4.50 | 2.80 | 3.10 | 3.90 | 2.90 | 3.20 | 4.60 | 1.20 | (2) |
| **Specific root length** | m/g | 23.90 | 64.50 | 57.80 | 90.30 | 74.00 | 41.30 | 48.30 | 39.50 | 68.30 | 16.10 | 71.90 | 13.90 | (2) |
| **Tree height** | cm | 131.8 | 233.0 | 235.0 | 260.7 | 414.0 | 341.0 | 129.6 | 145.6 | 127.4 | 165.2 | 304.0 | 154.9 | (2) |
| **Ground diameter** | cm | 27.6 | 34.4 | 28.5 | 31.1 | 43.0 | 43.0 | 28.6 | 34.6 | 25.5 | 32.0 | 34.8 | 31.2 | (2) |
| **Root nitrogen** | mg/g | 12.1 | 12.1 | 14.4 | 9.9 | 11.0 | 13.2 | 10.5 | 13.0 | 10.9 | 13.6 | 7.5 | 9.5 | (3) |
| **Root carbon** | mg/g | 487.7 | 485.7 | 482.9 | 498.9 | 497.4 | 492.6 | 495.5 | 490.2 | 495.2 | 482.9 | 498.0 | 496.8 | (3) |
| **Root phosphorus** | mg/g | 2.12 | 1.91 | 1.94 | 1.56 | 1.31 | 1.93 | 2.15 | 1.78 | 1.70 | 1.62 | 1.49 | 1.05 | (3) |
| **Root potassium** | mg/g | 1.84 | 1.99 | 1.89 | 1.52 | 1.45 | 1.48 | 1.77 | 1.24 | 1.54 | 1.16 | 2.16 | 1.23 | (3) |
| **Root calcium** | mg/g | 10.48 | 7.83 | 7.4 | 10.73 | 10.34 | 8.49 | 8.46 | 8.39 | 8.41 | 9.58 | 7.28 | 10.14 | (3) |
| **Root magnesium** | mg/g | 1.26 | 1.34 | 1.20 | 0.99 | 0.99 | 1.00 | 0.94 | 1.02 | 1.10 | 1.04 | 1.18 | 0.90 | (3) |
| **Wood density** | g/cm^3^ | 0.33 | 0.49 | 0.56 | 0.55 | 0.48 | 0.49 | 0.33 | 0.41 | 0.37 | 0.34 | 0.56 | 0.3 | (4) |
| **Seed mass** | g/1000 | 7.6 | 23.7 | 55.2 | 1 | 0.3 | 2 | 2.4 | 9 | 3.3 | 17 | 3143 | 1.5 | (5) |

**Table S2.** Mean values and units of tree functional traits used to compute functional diversity indices (FD_t_). Ab (*Abies balsamea*)*,*Ar (*Acer rubrum*)*,* As (*Acer saccharum*)*,* Ba (*Betula alleghaniensis*)*,* Bp (*Betula papyrifera*)*,* Ll (*Larix laricina*)*,* Pg (*Picea glauca*)*,* Pre (*Pinus resinosa*), Pru (*Picea rubens*)*,* Ps (*Pinus strobus*)*,* Qr (*Quercus rubra*)*,* To (*Thuja occidentalis*)*.* (1) *in situ* Jewell, M. (2014). MSc thesis, (2) *in situ* Tobner et al (2014). Frontiers (incl. unpublished), (3) *in situ* Khlifa et al. in preparation,, (4) Literature Chave, (5) Literature Kew.

|  |  |  |  |  |  |  |  |  |  |  |  |  |  |  |  |  |  |  |
| --- | --- | --- | --- | --- | --- | --- | --- | --- | --- | --- | --- | --- | --- | --- | --- | --- | --- | --- |

**Table S3.** Average values in (ng g^-1^) of total PLFA (PLFAtot) and PLFAs for different taxonomic groups fungi (PLFAfun), bacteria (PLFAbact), Gram positive (PLFApos) and negative bacteria (PLFAneg) for soils from plots of tree species mixtures (1,2,4 and 12 species) and their identities at the IDENT Montreal site (Québec, Canada). SR1 (monocultures), SR2 (two species mixtures), SR4 (four species mixtures) and SR12 (all twelve species together). Ab (*Abies balsamea*)*,* Ar (*Acer rubrum*)*,* As (*Acer saccharum*)*,* Ba (*Betula alleghaniensis*)*,* Bp (*Betula papyrifera*)*,* Ll (*Larix laricina*)*,* Pg (*Picea glauca*)*,* Pru (*Picea rubens*)*,* Pre (*Pinus resinosa*)*,* Ps (*Pinus strobus*)*,* Qr (*Quercus rubra*)*,* To (*Thuja occidentalis*)*.* In the case of monocultures, means with the same letter are not significantly different based on the Tukey-HSD post hoc test.

| **Treatment** | **SR**  **levels** | **FDclass**  **levels** | **PLFA_tot_** | | **PLFA_fun_** | **PLFA_bac_** | | | **PLFA_pos_** | | **PLFA_neg_** |
| --- | --- | --- | --- | --- | --- | --- | --- | --- | --- | --- | --- |
| **SR1** | 1 | 0 | 1995 |  | 141 | 918 |  | 535 | |  | 384 |
| **SR2** | 2 | - | 1997 |  | 140 | 896 |  | 521 | |  | 375 |
| **SR4** | 4 | - | 2055 |  | 131 | 835 |  | 454 | |  | 381 |
| **SR12** | 12 | - | 1730 |  | 178 | 846 |  | 416 | |  | 430 |
| **Ab** | 1 | 0 | 2017 | ab | 159 | 863 | ab | 511 | | ab | 353 |
| **Ar** | 1 | 0 | 2191 | ab | 199 | 1127 | ab | 591 | | ab | 537 |
| **As** | 1 | 0 | 2270 | ab | 125 | 1081 | ab | 674 | | ab | 407 |
| **Ba** | 1 | 0 | 1913 | ab | 157 | 888 | ab | 477 | | ab | 412 |
| **Bp** | 1 | 0 | 2034 | ab | 162 | 929 | ab | 536 | | ab | 393 |
| **Ll** | 1 | 0 | 2736 | a | 212 | 1329 | a | 795 | | a | 534 |
| **Pg** | 1 | 0 | 1673 | ab | 86 | 731 | ab | 452 | | b | 279 |
| **Pre** | 1 | 0 | 1815 | ab | 118 | 830 | ab | 490 | | ab | 340 |
| **Pru** | 1 | 0 | 1716 | ab | 115 | 758 | ab | 453 | | b | 305 |
| **Ps** | 1 | 0 | 2031 | ab | 146 | 877 | ab | 521 | | ab | 356 |
| **Qr** | 1 | 0 | 2120 | ab | 119 | 939 | ab | 522 | | ab | 416 |
| **To** | 1 | 0 | 1430 | b | 95 | 669 | b | 396 | | b | 273 |
| **PrePru** | 2 | 1 | 1590 |  | 86 | 699 |  | 414 | |  | 285 |
| **LlPs** | 2 | 2 | 2266 |  | 211 | 1054 |  | 629 | |  | 425 |
| **BaQr** | 2 | 3 | 1789 |  | 123 | 797 |  | 458 | |  | 339 |
| **BpQr** | 2 | 4 | 1982 |  | 190 | 975 |  | 510 | |  | 465 |
| **PgPs** | 2 | 5 | 2122 |  | 155 | 912 |  | 514 | |  | 398 |
| **AbAr** | 2 | 6 | 1725 |  | 72 | 777 |  | 525 | |  | 252 |
| **AsLl** | 2 | 7 | 1826 |  | 120 | 827 |  | 541 | |  | 287 |
| **AsTo** | 2 | 8 | 1940 |  | 113 | 838 |  | 509 | |  | 328 |
| **ArBa** | 2 | 2 | 1941 |  | 157 | 923 |  | 520 | |  | 403 |
| **PrePs** | 2 | 2 | 1876 |  | 131 | 878 |  | 504 | |  | 373 |
| **LlPg** | 2 | 4 | 2607 |  | 189 | 1128 |  | 627 | |  | 501 |
| **BpPs** | 2 | 5 | 2004 |  | 165 | 916 |  | 487 | |  | 430 |
| **AbAs** | 2 | 7 | 1765 |  | 89 | 763 |  | 472 | |  | 291 |
| **ArTo** | 2 | 7 | 2531 |  | 155 | 1064 |  | 584 | |  | 480 |
| **AbPgPrePru** | 4 | 1 | 1934 |  | 111 | 809 |  | 481 | |  | 328 |
| **ArBaBpQr** | 4 | 2 | 2206 |  | 107 | 782 |  | 407 | |  | 375 |
| **BaPrePruPs** | 4 | 3 | 2002 |  | 187 | 955 |  | 497 | |  | 459 |
| **AbBpLlPg** | 4 | 4 | 2235 |  | 115 | 866 |  | 495 | |  | 371 |
| **AbAsPruPg** | 4 | 5 | 1709 |  | 117 | 786 |  | 426 | |  | 360 |
| **LlPsQrTo** | 4 | 6 | 1654 |  | 86 | 803 |  | 436 | |  | 367 |
| **ArQrPsTo** | 4 | 7 | 1474 |  | 96 | 667 |  | 383 | |  | 283 |
| **AsBaPgTo** | 4 | 8 | 3268 |  | 174 | 1016 |  | 502 | |  | 514 |
| **PrePsPruPg** | 4 | 2 | 2134 |  | 141 | 821 |  | 478 | |  | 343 |
| **BpPsAsPg** | 4 | 7 | 1575 |  | 126 | 741 |  | 412 | |  | 329 |

**Table S4.** Model fit statistics and AICc index for the different functions describing the relationship between soil microbial parameters and tree species richness. Basal respiration(BR), active microbial biomass (GIR) expressed in µg C-CO_2_ g^−1^soil h^−1^ and metabolic quotient (qCO_2_) based on 4 replications for each identity plot for a total of 148 plots. AICc measures the relative goodness of fit of a given model; the lower its value, the more likely it is that this model is correct. Two models with ΔAICc > 2 are substantially different. The power function can fit multiple shapes and may represent either functional or no functional redundancy depending on each particular case.

| **Microbial parameter** | **Model** | **R^2^** | ***P*** | **AICc** | **Delta**  **AICc** | **Represented**  **Model** | **Model**  **group** |
| --- | --- | --- | --- | --- | --- | --- | --- |
| **Basal respiration (BR)** | Exponential^1^ | 0.071 | 0.001 | -283.640 | 16.265 |  |  |
|  | Linear^2^ | 0.080 | 0.004 | -285.268 | 14.637 |  |  |
|  | Power^3^ | 0.137 | <0.001 | -294.563 | 5.342 |  |  |
|  | Logarithmic^4^ | 0.147 | <0.001 | -296.172 | 3.733 |  |  |
|  | Michaelis-Menten^5^ | 0.168 | <0.001 | -299.905 | 0.000 | ✓ | No redundancy |
| **Active microbial biomass (GIR)** | Exponential^1^ | 0.001 | 0.650 | 204.571 | 3.729 |  |  |
|  | Linear^2^ | 0.001 | 0.623 | 204.536 | 3.694 |  |  |
|  | Power^3^ | 0.015 | 0.138 | 202.545 | 1.703 |  |  |
|  | Logarithmic^4^ | 0.016 | 0.122 | 202.356 | 1.514 |  |  |
|  | Michaelis-Menten^5^ | 0.026 | 0.049 | 200.842 | 0.000 | ✓ | No redundancy |
| **Metabolic quotient (qCO_2_)** | Exponential^1^ | 0.021 | 0.074 | -102.793 | 0.127 |  |  |
|  | Linear^2^ | 0.021 | 0.074 | -102.801 | 0.119 |  |  |
|  | Power^3^ | 0.024 | 0.069 | -102.918 | 0.002 |  |  |
|  | Logarithmic^4^ | 0.025 | 0.069 | -102.920 | 0.000 |  |  |
|  | Michaelis-Menten^5^ | 0.022 | 0.069 | -102.910 | 0.010 | ✓ | No redundancy |

**Model**

^1^ Y = a*exp(b*X)

^2^ Y = a*X + b

^3^ Y = a*X^b

^4^ Y = a + b · log(X)

^5^ Y = a*X/(b+X)
